# Glimpse into the future: harnessing autophagy to promote anti-tumor immunity with the DRibbles vaccine

**DOI:** 10.1186/s40425-016-0130-4

**Published:** 2016-05-17

**Authors:** David B. Page, Tyler W. Hulett, Traci L. Hilton, Hong-Ming Hu, Walter J. Urba, Bernard A. Fox

**Affiliations:** Earle A. Chiles Research Institute / Providence Portland Cancer Center, 4805 N.E. Glisan St., North Tower, Suite 2N87, Portland, OR 97213 USA; UbiVac, Inc., Portland, OR USA; Oregon Health & Science University, Portland, OR USA

**Keywords:** DRibbles, DPV-001, Autophagy, Immunotherapy, Vaccine, Autophagosome, Cross-presentation, Bortezomib

## Abstract

Because the benefits of immune checkpoint blockade may be restricted to tumors with pre-existing immune recognition, novel therapies that facilitate *de novo* immune activation are needed. DRibbles is a novel multi-valent vaccine that is created by disrupting degradation of intracellular proteins by the ubiquitin proteasome system. The DRibbles vaccine is comprised of autophagosome vesicles that are enriched with defective ribosomal products and short-lived proteins, known tumor-associated antigens, mediators of innate immunity, and surface markers that encourage phagocytosis and cross-presentation by antigen presenting cells. Here we summarize the rationale and preclinical development of DRibbles, translational evidence in support of DRibbles as a therapeutic strategy in humans, as well as recent developments and expected future directions of the DRibbles vaccine in the clinic.

## Background: cross-priming and the DRibbles vaccine

A successful anti-tumor immune response by cytotoxic CD8+ T cells requires recognition of tumor antigen in the context of MHCI molecules. One potential explanation for how naïve T-cells become activated against tumor antigens is a process called cross-presentation. During cross-presentation, professional antigen presenting cells (pAPCs) phagocytose tumor proteins, digest them with proteasomes, and present them via MHCI to T cells for activation. Two hypothesized classes of tumor-associated proteins—called defective ribosomal products (DRiPs) and short-lived proteins (SLiPs)— are produced in abundance within tumor cells, however are inherently unstable and only expressed transiently under physiologic conditions before being poly-ubiquitinated and degraded by tumor cell proteosomes [1]. These tumor-associated DRiPs/SLiPs, while expressed frequently on tumor MHCI, would be inefficiently cross-presented by pAPCs, possibly because they are degraded before they reach the APCs. It has been hypothesized that these DRiPs/SLiPs antigens, if delivered to pAPCs for cross-presentation, could potentially facilitate anti-tumor immune responses and could form the basis of a novel anti-tumor vaccine.

**Fig. 1:**
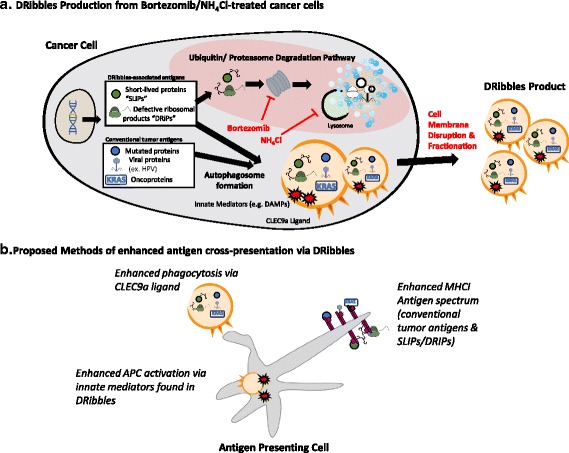
The DRibbles vaccine product is generated by manipulating the endogenous autophagy pathway, and is comprised of autophagosomes that contain antigens was well as mediators of innate immunity and phagocytosis

Here, we introduce the DRibbles vaccine product, which is produced by simultaneously blocking proteosomal degradation and manipulating the cellular autophagy pathway, leading to stabilization of DRiPs/SLiPs proteins and formation of autophagosome microvesicles that contain not only DRiPs/SLiPs, but also other protein products that have been shown to facilitate cross-presentation. These autophagosomes are then harvested by membrane disruption and fractionation to create the vaccine called DRibbles. Here, we summarize the preclinical data supporting the DRibbles vaccine, translational evidence in support of its efficacy in humans, and completed and ongoing clinical trials of DRibbles across a variety of malignancies.

## In the lab: preclinical development of the DRibbles vaccine

Evidence supporting the utility of the DRibbles concept for priming T cell responses was first demonstrated in a series of in vitro experiments using a modified OVA-expressing HEK 293 T tumor cell model [2]. The OVA gene was engineered to produce “short-lived” OVA proteins that would become poly-ubiquinated and degraded by proteasomes under physiologic conditions [2, 3]. Whole cells were treated with bortezomib (Velcade®, Takeda, Osaka, Japan) and ammonium chloride (NH_4_Cl), which block proteasome activity and lysosomal digestion of autophagosomes, respectively. Then, the treated cells were mechanically disrupted and fractionated by centrifugation to harvest an autophagosome-enriched product (Fig. 1a). This product was termed “DRibbles,” an acronym for “DRiPs and SLiPs-containing blebs.” The short-lived OVA proteins were found to be enriched in this DRibbles autophagosome product, compared to non-treated cells or non-disrupted bortezomib/NH_4_Cl-treated cells. Furthermore, DRibbles vaccine was superior in priming OVA-specific T cells compared to non-treated or non-disrupted cells. These data suggested that DRibbles could be an effective vaccine against endogenous tumor-associated short-lived proteins.

Next, the DRibbles vaccine was evaluated for in vivo efficacy. DRibbles can either be produced based on an autologous concept (i.e. making the vaccine from a patient’s own tumor) or an allogeneic concept (i.e. making an “off-the-shelf” vaccine from one or more tumors to be administered to many patients). To model the autologous concept, DRibbles vaccine was generated from a 3LL Lewis lung cancer cell line and was shown to delay tumor growth and improve survival in that cancer model [4]. Next, to model the allogeneic concept, DRibbles vaccine was generated from multiple implantable methylcholantherene (MCA)-induced sarcoma cell lines. The long-standing paradigm was that whole-cell MCA vaccine would be effective only against homologous tumors [5]. However, vaccination with DRibbles derived from unrelated MCA-induced sarcomas was also effective in slowing tumor growth of other, independently-derived MCA sarcomas [3]. T cells isolated from these mice released interferon gamma against both homologous and independently-derived tumors, suggesting they had been cross-primed to a broader array of antigens present across a variety of sarcomas. This phenomenon was called ‘cross-protection,’ and provided evidence that an allogeneic DRibbles vaccine might serve as an “off-the shelf” vaccine in the clinic.

Further work was conducted to characterize components of the DRibbles vaccine. It was confirmed in various cell lines that DRibbles contain long-lived proteins (i.e. proteins not destined for rapid poly-ubiquitination and degradation), and are enriched for short-lived proteins, short-protein fragments, and poly-ubiquitinated proteins [4]. In addition to these potential antigens, the murine DRibbles product contained various damage-associated molecular pattern (DAMP) signals including heat shock proteins, high-mobility group box 1 protein (HMGB1) and calreticulin, suggesting that DRibbles could potentially mediate both adaptive and innate immunity. Finally, the DRibbles autophagosome surfaces were found to contain CLEC9A ligands, which have been shown to bind CLEC9A receptor [6] and facilitate antigen uptake by a subset of dendritic cells that play an important role in cross-presentation [7] (Fig. 1b). In summary, DRibbles was found to be composed of microvesicles that efficiently deliver a variety of antigens to pAPCs in ways that traditional liposomal and cellular vaccines do not.

## Bench to bedside: translational data regarding the DRibbles vaccine

The results of this characterization, combined with the promise of ‘cross-protection’, led to the development of various human DRibbles autophagosome vaccine formulations for the treatment of human subjects. The first allogeneic human DRibbles vaccine, named DPV-001, was derived from autophagosome products of two human cancer cell lines: UbiLT3 and UbiLT6. UbiLT3 was derived from a non-small cell lung carcinoma (NSCLC) of mixed histology, whereas UBiLT6 was derived from an NSCLC adenocarcinoma. Liquid chromatography tandem mass spectrometry and western blotting techniques have been used to quantitatively catalogue over 2400 of the most common protein constituents in DPV-001. Of these most common proteins, there are over 25 published cancer-associated antigens, including at least 12 proteins that are among the NCI’s list of prioritized cancer antigens [8] such as TP53, survivin, EphA2, cyclin B1, XAGE1, Her2/neu, RhoC, Mesothelin, Legumain, PDGFRb, FOSL1 and KRAS [9].

Whole exome sequencing was used to show that many of the UBiLT3/6 genes are mutated or polymorphic versus the reference human genome (hg19). Thus, the DPV-001 DRibbles vaccine likely contains protein variants that are foreign to vaccinated patients. The UbiLT3/6 sequences were compared to 520 unique lung adenocarcinoma sequences from The Cancer Genome Atlas (TCGA) [10]. In addition to containing commonly observed oncogene mutations (for example KRAS G12C, found in 6.8 % of adenocarcinomas in TCGA, http://www.cbioportal.org/index.do, accessed February 6, 2016), the UbiLT3/6 cell lines also shared polymorphisms with the identified non-synonymous mutations from each lung adenocarcinoma in the TCGA. This suggests that DRibbles may serve as an off-the-shelf vaccine against “private antigens” found in individual patients. Furthermore, non-exact foreign protein variants (for example other KRAS G12 codon point mutations) may function as altered-peptide ligands which stimulate immune responses that spread to a patient’s own tumor-specific neo-epitopes [11, 12].

## In the clinic: development of human DRibbles vaccines

In humans, the DRibbles vaccine was first evaluated as an autologous vaccine manufactured with tumor cells isolated from pleural effusions of patients with NSCLC. In this phase I clinical trial, autologous DRibbles vaccine was found to be safe when combined with docetaxel plus GM-CSF [13]. Autologous DRibbles vaccines, while providing a potential opportunity to vaccinate against patient-specific neo-epitopes, have proven difficult to manufacture consistently. Furthermore, a recent study suggested that in melanoma patients, CD8+ T cells may more frequently recognize non-mutated antigens such as NY-ESO-1 and GP100, rather than neoepitopes [14]. Subsequent trials have focused on allogeneic DRibbles products, which contain numerous non-mutated self-antigens. Studies are underway to evaluate the role of allogeneic DRibbles in malignancies such as prostate adenocarcinoma and NSCLC (Table 1). These studies are also assessing the DRibbles vaccine in conjunction with low-dose cyclophosphamide and various adjuvants such as topical imiquimod or GM-CSF.

**Table 1:** Summary of preclinical, translational, and clinical evidence of the DRibbles vaccine

In the Lab	Bench to Bedside	In the Clinic
• DRibbles vaccine comprises autophagosome-packaged cellular proteins, short-lived proteins and ribosomal proteins that may be missed by endogenous immunity	• The DPV-001 DRibbles contains over 2,000 proteins, of which 25 are known tumor-associated antigens	• Dribbles vaccine + docetaxel was well tolerated in a phase I NSCLC trial
• DRibbles delays growth in both “autologous” and “allogeneic” pre-preclinical models	• The DPV-001 cell lines contain thousands of mutations and polymorphisms that could function as altered-peptide ligands	• A Phase II trial comparing DRibbles + either GM-CSF or Imiquimod in stage IIIA/B NSCLC is ongoing
• DRibbles contains innate immunity mediators (DAMPs) and surface ligands for CLEC9a, which facilitate uptake by APCs for cross-presentation	• Each lung adenocarcinoma sequence from the TCGA database shares at least one mutation with the polymorphisms found in DPV-001	• A Phase I trial of DRibbles + imiquimod + low-dose cyclophosphamide is ongoing
	• DPV-001 contains common oncogene mutants such as KRAS G12C	• DRibbles induced increased antibody reactivity in the first 2 patients treated on a phase II trial

In addition to DRibbles, a multitude of clinical trials are underway evaluating safety and anti-tumor efficacy of other modulators of autophagy such as hydroxychloroquine and alpha-tocopheryloxyacetic acid (alpha-TEA) [15–17]. Besides DRibbles, there are reports of alternative potentially effective cell-derived vesicle vaccines. For example, melanoma patients have been treated with an autologous product of dendritic cell-derived exosomes pulsed with tumor antigen peptides [18]. More recently, another group has shown that tumor-derived exosomes could be a more effective method for priming anti-tumor immune responses compared to tumor lysate alone [19].

One major barrier for all cancer vaccine clinical trials is the difficulty in demonstrating efficacy in early stage disease, especially in tumor types with low recurrence rates or prolonged latency periods. Therefore, scientifically grounded immune monitoring strategies must be used to inform early development of vaccines, allowing for smaller trials designed to facilitate optimization of the vaccine and perhaps identify indirect evidence of efficacy. Because DRibbles vaccines are multi-valent, and because relevant antigenic targets may vary across patients, next-generation high-throughput technologies such as seromic protein arrays are being used to evaluate patient-specific immune responses and discover relevant antigens. The phase II non-small cell lung cancer DRibbles adjuvant trial serves as an example of how a seromics-based approach might be used to monitor immune response. This trial was designed to detect enhancement in antigen-specific adaptive immunity using serum protein arrays that measure antibody reactivity against a panel of over 8,000 normal human protein isoforms. The rationale is that the most robust immune responses might be integrated with concomitant CD4 T-helper, CD8 cytotoxic, and humoral immune responses [20], and therefore antibody reactivity may serve to identify antigen-specific immune responses associated with therapy. Using the protein array, several of the 9 treated DRibbles patients were found to exhibit robust (i.e. >10-fold increase from baseline) antibody responses to multiple antigens following vaccination [10].

## Conclusion

The DRibbles vaccine serves as an excellent example of how basic immunology research can be translated into a promising approach in the clinic. Because the DRibbles platform can be used to generate either autologous or allogeneic vaccines derived from any tumor cell line, it may have clinical applications across a broad range of malignancy types. Relative to peptide and DNA vaccines, the DRibbles autophagosome-enriched vaccine platform may serve to vaccinate broadly against a spectrum of antigen types including potential neo-epitopes and a short-lived/defective cellular proteins that may not be present in other complex cell-derived cancer vaccines. Additionally, molecules such as CLEC9a ligand encourage uptake of DRibbles by cross-presenting pAPCs—a property not present in traditional liposome or microvesicle vaccine formulations. Because of these unique features, the DRibbles construct may in the future be investigated as a delivery mechanism for other vaccines, such as personalized patient-specific cancer neo-epitope peptides.

## Ethics approval and consent to participate

Not applicable.

## Abbreviations

Alpha-TEA: Alpha-tocopheryloxyacetic acid
DAMP: Damage-associated molecular pattern
DRiPs: Defective ribosomal products
HMGB1: High-mobility group box 1
MCA: Methylcholantherene
NH_4_Cl: Ammonium chloride
NSCLC: Non-small cell lung carcinoma
pAPC: Professional antigen presenting cell
SLiPs: Short-lived proteins
TCGA: The Cancer Genome Atlas
